# Tumor-Infiltrating B- and T-Cell Repertoire in Pancreatic Cancer Associated With Host and Tumor Features

**DOI:** 10.3389/fimmu.2021.730746

**Published:** 2021-09-23

**Authors:** Silvia Pineda, Evangelina López de Maturana, Katharine Yu, Akshay Ravoor, Inés Wood, Núria Malats, Marina Sirota

**Affiliations:** ^1^ Genetic and Molecular Epidemiology Group, Spanish National Cancer Research Centre (CNIO), and Centro de Investigación Biomédica en Red Cáncer (CIBERONC), Madrid, Spain; ^2^ Bakar Computational Health Sciences Institute, University of San Francisco, California (UCSF), San Francisco, CA, United States; ^3^ Department of Pediatrics, University of San Francisco, California (UCSF), San Francisco, CA, United States

**Keywords:** B-cell repertoire, immunoglobulins, T-cell repertoire, pancreatic cancer, tumor microenvironment, tumor infiltration, compositional analysis

## Abstract

**Background:**

Infiltrating B and T cells have been observed in several tumor tissues, including pancreatic ductal adenocarcinoma (PDAC). The majority known PDAC risk factors point to a chronic inflammatory process leading to different forms of immunological infiltration. Understanding pancreatic tumor infiltration may lead to improved knowledge of this devastating disease.

**Methods:**

We extracted the immunoglobulins (IGs) and T cell receptors (TCRs) from RNA-sequencing of 144 PDAC from TCGA and 180 pancreatic normal tissue from GTEx. We used Shannon entropy to find differences in IG/TCR diversity. We performed a clonotype analysis considering the IG clone definition (same V and J segments, same CDR3 length, and 90% nucleotide identity between CDR3s) to study differences among the tumor samples. Finally, we performed an association analysis to find host and tumor factors associated with the IG/TCR.

**Results:**

PDAC presented a richer and more diverse IG and TCR infiltration than normal pancreatic tissue. A higher IG infiltration was present in heavy smokers and females and it was associated with better overall survival. In addition, specific IG clonotypes classified samples with better prognosis explaining 24% of the prognosis phenotypic variance. On the other hand, a larger TCR infiltration was present in patients with previous history of diabetes and was associated with lower nonantigen load.

**Conclusions:**

Our findings support PDAC subtyping according to its immune repertoire landscape with a potential impact on the understanding of the inflammatory basis of PDAC risk factors as well as the design of treatment options and prognosis monitoring.

## Introduction

Pancreatic ductal adenocarcinoma (PDAC) is a dreadful disease and, despite its relatively low population incidence, it is the deadliest cancer worldwide with a 7% 5-year survival rate. It is projected that PDAC will become second in the cancer mortality ranking before 2030 (1) if action is not immediately taken. Furthermore, this is the single cancer for which there has been no improvement in its fatal prognosis over the last decades despite the many efforts to improve tertiary prevention (treatment). Even new personalized treatments, through more radical surgical resection, neoadjuvant, immunotherapy, and targeted chemotherapy, benefits only a small fraction of patients.

PDAC is a multifactorial and heterogeneous disease. To decipher its complexity at both molecular and etiological level, more comprehensive strategies are needed. Among the well-established PDAC risk factors, tobacco, alcohol, non-O blood group, chronic pancreatitis, type 2 diabetes mellitus, and obesity have been associated with an increased risk of PDAC (2–4), while nasal allergies and asthma were associated with reduced risk (5). At the molecular level, somatic copy number alterations and mutations leading to altered expression of key oncogenes and tumor suppressor genes (KRAS, TP53, SMAF4, and CDKN2A) contribute to the complex molecular landscape of PDAC (6–8). Despite these important findings, there are still a number of unknown risk factors, probably interacting with molecular factors, contributing to this devastating disease.

The majority of known risk factors point to a chronic inflammatory process and different forms of inflammation play critical roles at different stages of tumor development, which might result in tumor microenvironments containing innate immune cells (macrophages, neutrophils, and mast, myeloid-derived suppressor, dendritic, and natural killer cells) and adaptive immune cells (T and B lymphocytes), in addition to cancer cells and their surrounding stroma (fibroblasts, endothelial cells, pericytes, and mesenchymal cells). Furthermore, there is a strong intercommunication among all these components either by direct contact or by production of cytokines and chemokines controlling the tumor growth (9). Indeed, six immune subtypes based on immune expression signatures across all TCGA tumors have been proposed and showed an association with prognosis, genetics, and immune modulatory alterations, demonstrating the importance of studying the immune infiltration as an important factor in cancer development (10), as already shown in PDAC (11–13), as well as in other cancers (14). The microenvironment of human cancer is complex and often shows different characteristics according to the carcinogenic pathways involved, the mutations harboring neo-antigens, or their clinicopathological impact interacting with the adaptive immune system that acts as orchestrator and effector of immunity. Therefore, a characterization of the tumor immune-infiltrating cells of the adaptive immune response can be of potential importance.

The key feature of B and T cells is their enormous diversity. A potent adaptive immune response is reliant upon the expansion of B- and T-cell clones during infection. In the context of PDAC, the T-cell immune repertoire (IR) has been characterized in peripheral blood (15–17) and, while T-cell infiltration has been observed in several tumor tissues, the function of infiltrating B cells is still ill-defined (18). Therefore, the aim of this study was to characterize the tumor-infiltrating B- and T-cell repertoire in PDAC and its interaction with host and tumor features. We found that the PDAC microenvironment presented significantly higher TCR and IG infiltration than normal pancreatic tissue. We also found that TCRs were associated with the lower presence of neoantigens and previous history of diabetes while the IGs were associated with better prognosis and were higher in females and heavy smokers. Selected IGK clones classified PDAC tumors with better prognosis and the variability of IGK clones explained 24% of the prognostic phenotypic variance. These results support PDAC subtyping according to its IR landscape with a potential impact on understanding the inflammatory basis of PDAC risk factors as well as the design of treatment options and prognosis improvement.

## Materials and Methods

### TCGA Data

We downloaded the RNAseq fastq file with a total of 181 samples from PAAD-TCGA from the legacy. We used TCGA biolinks to download the clinical and biospecimen samples (https://bioconductor.org/packages/release/bioc/html/TCGAbiolinks.html). We then used the samples from (19) for re-classification and restricted to those confirmed as PDAC, ending with a total of 144 patients. In comparison to previous analysis on PDAC samples from TCGA, we curated and extracted very carefully the PDAC samples.

### GTEx Data

We downloaded the RNAseq fastq files with a total 195 pancreas samples from dbGaP Accession phs000424 using the SRA run selector NCBI. Then, we filtered out those samples that did not pass QC for RNAseq as reported by the GTEx Consortium (20) and those that reported to have cancer. Finally, we used a final dataset with 180 pancreatic samples from healthy individuals.

### IG and TCR Data Extraction

We used MiXCR tool to align the RNA-seq fastq files to the VDJ region to extract IGH, IGK, and IGL, and TRA, TRB, TRD, and TRG. We applied the pipeline described in https://mixcr.readthedocs.io/en/master/ for alignments using paired-end RNA-seq. The median number of reads and clones for the four datasets are in **Supplementary Table S1**. TRD and TRG reads and clones were very low; therefore, we decided to filter out these receptors for the analysis. We performed a quality control procedure at sample level, filtering out the individuals with IG clones < 100 and those with T clones < 100, resulting in 143 and 135 samples, respectively.

### Diversity Analysis

We performed diversity analysis considering two measures. As the number of reads might be dependent on the total sequencing reads, we defined expression of IG and TCR as: ${IG}_{i}/{TCR}_{i}=\frac{M_{i}}{N_{i}+M_{i}},$ where *i* corresponds to each sample, *M_i_* is the number of reads that map to a specific IG/TCR, and *N_i_* is the number of reads that map to anything else in the genome.

We defined an IG clone as those reads that had the same V and J gene, same CDR3 length, and 90% of nucleotide identity, and a TCR clone as those reads that had the same V, J gene, and CDR3 length and 95% of nucleotide identity. We restricted this analysis to those reads that estimated the CDR3 region. This definition allowed studying diversity, shared and common clones, and clonal expansion. To define diversity, we used Shannon entropy (*H*), which provides information about the size distribution.


$$H=-\sum_{i=1}^{N}p_{i}{log}_{2}p_{i}$$

Where *N* is the number of unique clones and *p_i_* is the frequency of clone *i*. *H* ranges from 0 (sample with only one clone) to *Hmax* = *log*_2_ *N* (sample with a uniform distribution of clones).

Then, we used Wilcoxon rank test to measure the differences regarding the diversity and richness of all the features (IGH, IGK, IGL, TRA, and TRB expression and entropy) between the tumor and normal pancreatic tissue of four datasets (TCGA, GTEx, and the two validation sets). We also performed a Spearman correlation test to check the correlation among all the features considering both measures, richness and diversity, and all receptors (IGH, IGK, IGL, TRA, and TRB) in the TCGA and GTEx datasets.

The measures can be highly dependent on the sequencing depth. In the case of the expression, we accounted for this calculating the expression by dividing the number of reads by the total number of sequencing reads in the RNA-seq fastq files. For the entropy, although this measure should not be highly affected by the sequencing depth, we randomly sampled different proportions of the sequence data for each sample and, then, we calculated the entropy measure in its corresponding sampled dataset. We performed the random sampling 10 times in each proportion to avoid possible stochastic effects and calculated the mean value as the final estimate.

### Network Analysis

The network generation algorithm is similar to the one used previously by our group (21) and others (22). Briefly, each vertex represented a B/T-cell sequence where its size was defined by all the identical sequences. Edges were calculated using the clone definition (same V and J segments, same CDR3 length, and 90%/95% nucleotide identity between CDR3s for BCR/TCR, respectively) and clusters represent each clone in the repertoire. The analysis was done using igraph package in R using the layout_with_graphopt option to generate the plot.

To quantify the network, we calculated the Gini Index for vertex and cluster sizes. Gini Index was a measure of unevenness extensively used to measure wealth distribution. It measured the inequality among values of frequency distribution. We used the Gini function from ineq package in R to calculate the Gini coefficient for vertex size and cluster size distribution. A Gini coefficient of zero expresses perfect equality and a Gini coefficient of 1 expressed maximal inequality. When applied to vertex size, Gini(V), the overall clonal nature is represented. If Gini(V) was closer to 1, vertices were unequal, showing expansion of some of them, and closer to 0, otherwise. When applied to cluster size, Gini(C), clonal dominance was represented. If closer to 1, clusters were unequal and therefore represented dominant clones; if closer to 0, all clusters were of equal size.

### Association Analysis

To perform the association analyses between the immune features estimated in TCGA with the molecular data, immune characteristics, and the host and clinical data, we used Pearson correlation for the continuous variables and Wilcoxon test for the categorical variables. Since the expression features follow a skewed distribution, we applied a log10 transformation before performing the statistical test. The variables we analyzed were the ones reported in (23) considering tumor purity measured using ABSOLUTE (7, 24) inferring the purity based on DNA somatic mutations and leukocyte fraction based on DNA methylation as described by Carter et al. (24). We also assessed the association with the three classifications of PDAC subtypes (23). Then, we analyzed all the immune signatures proposed by Thorsson et al. (10) and all the clinical and epidemiological data available in the TCGA. Finally, we performed a survival analysis using Cox regression adjusted for age, sex, and pathological stage.

### Clonotype Analysis

Clonotypes were quantified as the number of reads for each clone per sample. The data produced after this definition involved high-dimensional structured multivariate and sparse data that are compositional. A composition is defined as a vector of positive real numbers, *x* = (*x*1,…,*xk*), *xi* > 0, that contains relative information. In addition, they were constrained by the sequencing depth that induces strong dependencies and spurious correlations among the number of reads for the different clones. To deal with all these issues, we took advantage of the compositional data analysis. Since we wanted to select a group of clonotypes associated with the outcome, we applied the CLR-LASSO to perform variable selection (25) using compositional data. This LASSO method transforms the data with the CLR transformation, which projects the compositional data to a Euclidean space. The clr transformation is defined as:


$$clr(x)=clr(x1,...,xk)=\left(\text{log}(\frac{x1}{g(x)}),...,\text{log}(\frac{xk}{g(x)})\right),$$

Where *g*(*x*) = (∏*x_j_*)^1/^ *^k^* is the geometric mean of the composition.

Before applying the CLR transformation, we added an offset of 1 to the whole matrix to deal with the zeros. So, we applied the CLR-LASSO to find the clonotypes of the IGH, IGK, and IGL associated differently with the normal pancreatic tissue (GTEx) and PDAC samples (TCGA). Then, we applied a hierarchical clustering with the clonotypes selected to cluster the samples. These results were validated by applying a principal component analysis using the four datasets (TCGA, GTEx, and the two validation sets). We also checked whether the samples grouped in the different clusters had different cell composition, which was obtained applying the xCELL tool (26) to the RNA-seq data, using a Kruskal–Wallis test.

### Estimation of Whole IGK Profile-Based Prognostic Score

We applied a Bayesian RKHS (27) to estimate the prognostic score for PDAC cases in TCGA based on their IGK profile:


y=Xβ+Zp+ϵ

Where **y** corresponds to the survival time of each PDAC case, x is an incidence matrix relating the systematic effects (β, age at initial pathologic diagnosis, gender, and pathologic stage) to each individual, Z is a design matrix allocating records (*y*) to the vector of whole IGK-based prognostic scores (p), and ϵ corresponds to the residual effects. p and ϵ are assumed to follow the prior distributions $p∼N(0,{\text{K}}_{IGK}\sigma_{\text{IGK}}^{2}$ ) and $\epsilon ∼\text{N}(0,\text{I}\sigma_{\epsilon }^{2}$ ), where K*_IGK_* is the IGK profile-based relationship matrix between individuals, computed using a linear kernel with the linkernel function of apcluster R package, and $\sigma_{\text{IGK}}^{2}$ is the the variance explained by the IGK profiles. The prior distributions for both variances ( $\sigma_{\text{IGK}}^{2}$ and $\sigma_{\epsilon }^{2}$ ) were inverted chi-square distributions.

Estimates of the systematic (β) and IGK-based prognostic scores (p) and those of the variances were obtained from their posterior distributions using a Gibbs sampling implemented in the BGLR *R* package (28). We ran a McMC chain of 500,000 iterations, and the first 100,000 were discarded as burn-in.

The proportion of the prognostic variance explained by the IGK-based variance was estimated as $\frac{\sigma_{IGK}^{2}}{\sigma_{IGK}^{2}+\sigma_{\epsilon }^{2}}$ .

## Results

### Study Subjects

**Figure 1:**
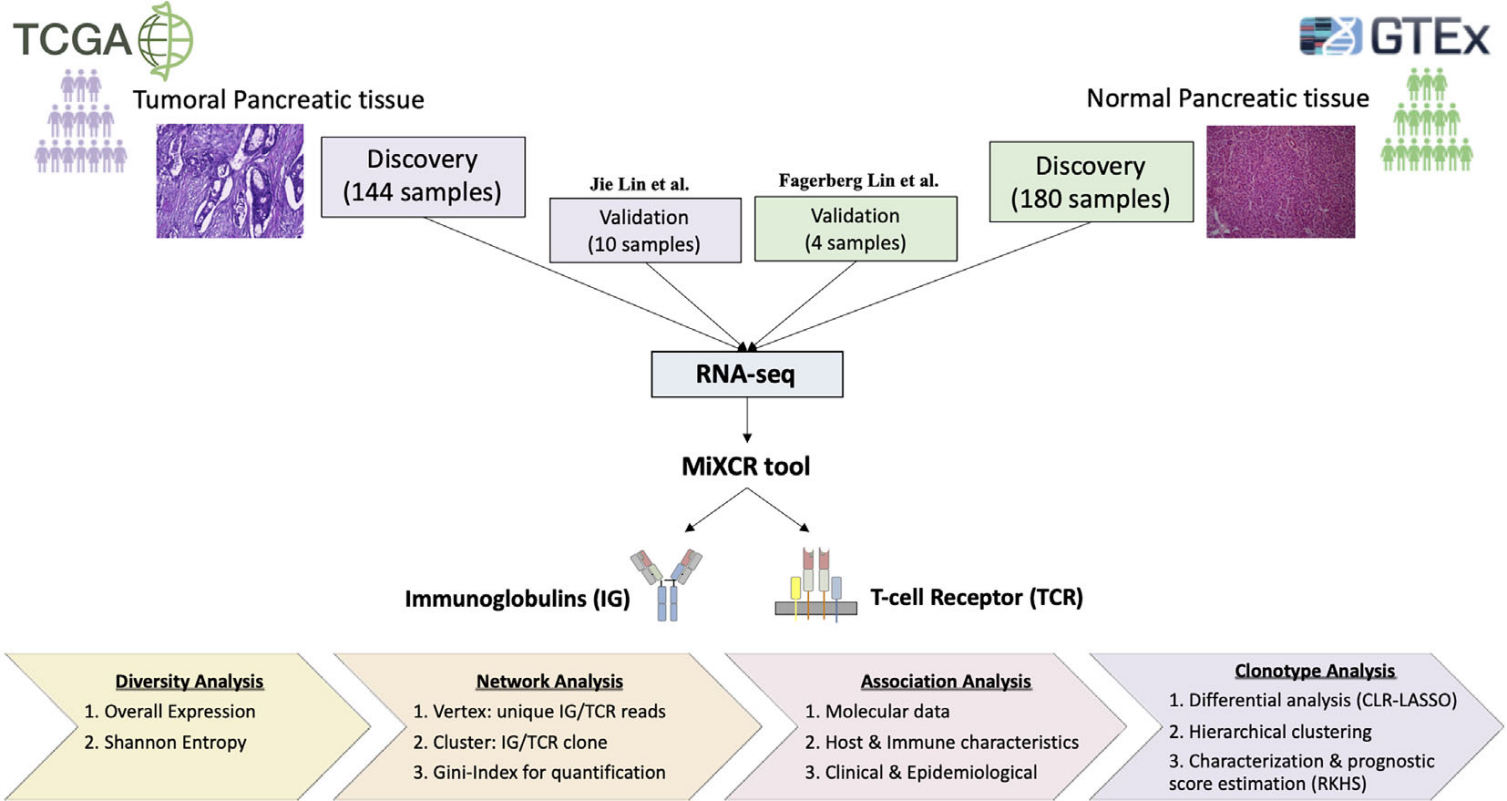
Overall study pipeline. Comparison of 144 tumor pancreatic tissue from TCGA with 180 normal pancreatic tissue from GTEx. Data for validation were considered from Jin et al. (29) with 10 PDAC samples and Fagerberg Lin et al. (30) with two replicated from two normal samples. We used the MiXCR tool to extract IG and TCR from RNA sequencing data and then performed a detailed statistical study to characterize properly the tumor-infiltrating immune repertoire by applying diversity, network, association, and clonotype analyses.

We used data from a total of 144 confirmed PDAC cases from TCGA (19) (https://portal.gdc.cancer.gov/) to extract the IG and TCR read sequences and characterize the tumoral immune infiltration using the proposed pipeline (**Figure 1**). We compared them with normal pancreatic tissue samples from 180 healthy individuals from GTEx (https://www.gtexportal.org/home/). Subject characteristics are shown in **Table 1**. The sex distribution was similar in both populations, but individuals with PDAC were significantly older (*p* < 0.001). To further validate our results, we used 10 PDAC samples publicly available from (29) and two pancreatic tissue samples with two replicates per sample from healthy individuals from (30). The PDAC samples from Jie Lin et al. had tumors with lower stage (*p* = 0.006) in comparison to the TCGA and were younger than the TCGA PDAC cases (*p* = 0.03).

**Table 1:** Patient/sample characteristics.

		TCGA (PDAC)	Jie Lin et al. (29) (PDAC)	^*^ *p*-value	GTEx (Normal)	^**^ *p*-value	Fagerberg Lin et al. (30) (Normal)
	PDAC Healthy	144	10		180		4
**Age**	Mean (SD)	64.9 (11.0)	61.4 (8.1)	0.03	50.4 (11.6)	<0.001	
**Sex**	Female	67 (46%)	4 (40%)	0.7	72 (40%)	0.3	
	Male	77 (54%)	6 (60%)		108 (60%)		
**Histological grade**	G1	20	1	0.06			
	G2	80	9				
	G3	43	0				
	Missing	1	0				
**Pathological stage**	Stage I	11	5	0.006			
	Stage II	124	5				
	Stage III	3	0				
	Stage IV	4	0				
	Missing	2	0				

^*^Comparison between TCGA (PDAC) and Jie Lin et al. (PDAC) using Fisher exact test for the categorical variables and Wilcoxon test for continuous variables.

^**^Comparison between TCGA (PDAC) and GTEx (Normal) using Fisher exact test for the categorical variables and Wilcoxon test for continuous variables.

### B- and T-Cell Repertoire

We used MiXCR (31) to extract the read sequences from RNA-seq to their respective IGH, IGK, and IGL, and TRA, TRB, TRD, and TRG types. We obtained a total number of 8,660,640 IG reads (mean 60,564/sample) and 92,153 TCR reads (mean 644/sample) for the TCGA samples and 227,011 IG reads (mean 1,261/sample) and 12,961 TCR reads (mean 72/sample) for the GTEx samples. **Supplementary Table S1** shows the sequencing summary for IG and TCR reads for all datasets. Then, we grouped the reads into clones (same V and J gene, same CDR3 length, and 90% for IG and 95% for TCR nucleotide identity) defined by those cells that come from the same common ancestor.

### Richness and Diversity of IG and TCR Infiltration Were Higher in PDAC Samples Compared to Normal Pancreatic Tissue Samples

**Figure 2:**
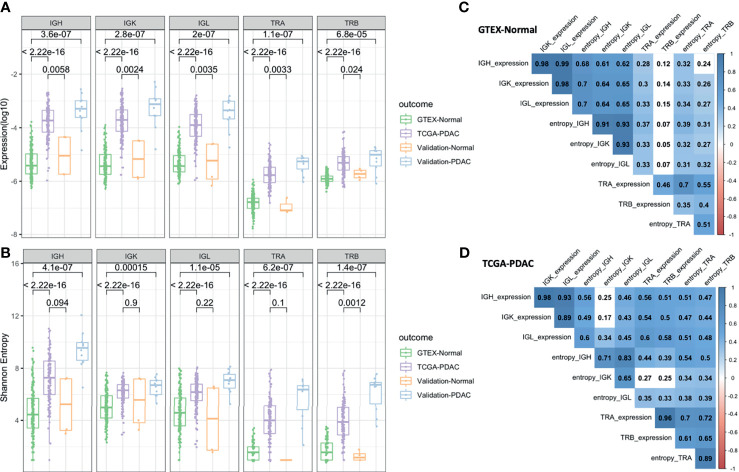
Diversity analysis. Boxplots showing IGH, IGK, IGL, TRA, and TRB expression **(A)** and Shannon entropy **(B)**. The *p*-values correspond to the statistical differences by applying a Wilcoxon test. Correlation plot for the GTEx-normal dataset **(C)** and TCGA-PDAC dataset **(D)**. The numbers correspond to the Pearson coefficient and the colored ones are statistically significant (FDR < 0.05).

We defined richness and diversity features to study the IR for all IG and TCR chain types. We accounted for richness using a measure of overall expression by counting the number of mRNA reads that map to an IG or TCR normalized by the total number of reads. Then, we calculated the diversity measured by the Shannon entropy to consider not only the number but also the size distribution of the clones. We observed a much higher IGH, IGK, IGL, TRA, and TRB richness and diversity in tumoral samples in comparison to the normal samples (*p* < 2.2 × 10^16^) in the discovery datasets (TCGA and GTEx). When compared with the validation set, the PDAC samples locate in the same ranges than the PDAC from TCGA showing highly significant *p*-values (*p* < 10^−5^) for all IG and TCR richness in comparison to GTEx. Similar results can be observed with the normal samples, although due to a very small sample size, the *p*-values were slightly significant, especially for the diversity measure (**Figures 2A, B**). We observed that richness and diversity of TCR were lower than those for IG [mean (TCR) = 644, mean (IG) = 60,563, *p* < 2.2 × 10^16^] (**Figures 2A, B**), although the IG and TCR richness was significantly correlated, especially in the PDAC samples (**Figures 2C, D**). As expected, we also observed that diversity of the IGL and IGK was lower than that of the IGH (*p* = 1.7 × 10^−10^) for PDAC samples (**Figure 2B**). To discard the effect of diabetes given that it is an immune-related disease and a risk factor of pancreatic cancer, we performed the same analysis in a subset of non-diabetic patients and the results remained significant (**Supplementary Figure S1**).

It is worth mentioning that the IGK richness was not significantly correlated (rho = 0.17) with its corresponding diversity in tumors (**Figure 2D**), while in normal tissue, the correlation was high (rho = 0.64) (**Figure 2C**). This could indicate that IGK clonotypes were more clonally expanded and had a more restricted IR in tumors. In an opposite direction, the correlation between the IG measures and TCR measures was lower in normal tissue and non-significant for TRB richness in comparison to PDAC.

An important requirement of this approach is that the measures must not be highly dependent on the depth of sequencing and scale invariant. In the case of the expression measures, we corrected each sample for its sequencing depth, and for the entropy measure, we tested all of the measures as a function of sequencing depth by doing a sensitivity analysis, consisting of randomly sampling different proportions of the sequence data for each sample. Then, we calculated the corresponding entropy parameter. Importantly, the entropy measures for all the IG and TCR types showed little variation at different sample sizes, even when subsampling was as low as 20% (**Supplementary Figure S2**).

### IG Repertoires Were Clonally Expanded in PDAC Samples While TCR Were Not

**Figure 3:**
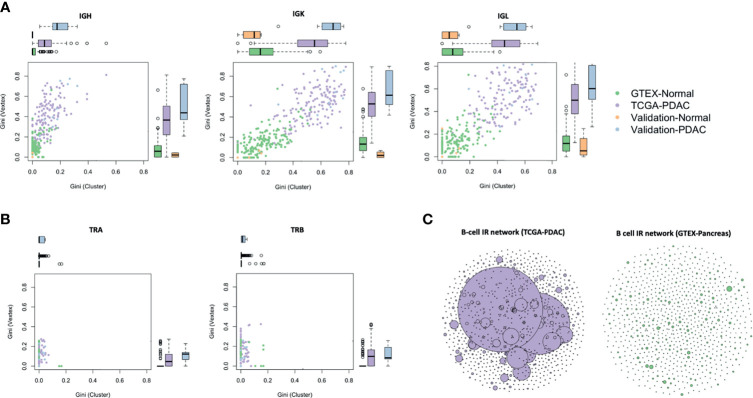
Network analysis. Vertex Gini Index plotted against Cluster Gini Index for IGH, IGK, and IGL **(A)**, and TRA and TRB **(B)**. The scatter plot represents each sample. Boxplots show the *Gini(V)* and *Gini(C)* differences. *p*-values (TCGA *vs*. GTEx) − IGH: *p (Gini(V*) < 2.2 × 10^−16^, *p* (*Gini(C) TCGA vs*. *GTEx*) < 2.2 × 10^−16^; IGK: *p (Gini(V*) < 2.2 × 10^−16^, *p* (*Gini(C) TCGA *vs*. GTEx*) < 2.2 × 10^−16^; IGL: *p (Gini(V*) < 2.2 × 10^−16^, *p* (*Gini(C) TCGA vs*. *GTEx*) < 2.2 × 10^−16^. B-cell repertoire networks **(C)** from two samples representing one PDAC from TCGA (purple) and one normal pancreas from GTEx (green). Each vertex represents a unique BCR being the vertex size defined by the number of identical BCRs considering the nucleotide sequences. An edge exists between vertices when they belong to the same clone as defined before, so clusters are groups of interconnected vertices forming a clone.

We defined an IG and TCR network based on sequence diversity as previously published by our group (21) and others (22). Each vertex was represented by a unique B- or T-cell receptor, and the number of identical B- or T-cell receptors based on their nucleotide sequences defined the vertex size. An edge was defined between vertices when they belonged to the same clone (same V and J gene, same CDR3 length, and 90% for IG and 95% for TCR nucleotide identity), so clusters of B or T cells could be seen as groups of interconnected vertices forming a clone. In order to compare the networks across samples, we used the Gini index, an unevenness measure ranging 0–1. When applied to vertex size (Gini(V)), the overall clonal nature was represented, meaning that if Gini(V) was closer to 1, vertices were unequally showing expansion of some of them, and closer to 0, otherwise. When applied to cluster size (Gini(C)), clonal dominance was represented. If G(C) was closer to 1, clusters were unequal and therefore they represented dominant clones; if closer to 0, all clusters were of equal size. We observed that PDAC samples had a much higher IGH, IGK, and IGL clonal expansion and more dominant clones in comparison to the normal samples in the discovery set (*p* < 2.2 × 10^16^), and similarly as before, this is validated in the other two datasets (**Figure 3A**). A network representation of the IG IR of one PDAC sample and one normal sample is represented in **Figure 3C**. While IGK and IGL showed very high Gini(C) [mean (sd) = 0.5 (0.1) and 0.4 (0.1), respectively] and Gini(V) [mean (sd) = 0.5 (0.2) and 0.5 (0.2), respectively] for PDAC samples, IGH had the lowest Gini(V) Gini(C) [mean (sd) = 0.3 (0.2) and 0.1 (0.1), respectively], meaning that the IGK and IGL IR might be more restricted by specific clones, likely because they were less diverse as we observed previously. Interestingly, we also found that the networks for TCR were not expanded or dominated by any clone (**Figure 3B**), suggesting that the T-cell repertoire was not as active as the B-cell repertoire in PDAC.

We performed a sensitivity analysis to test whether the Gini(C) and Gini(V) stayed invariant to the sequencing depth and we also showed little variation at different sample sizes, even when subsampling as low as 20% (**Supplementary Figure S3**).

### Immune Repertoire Features Were Significantly Associated With Other Molecular and Immune Characteristics of the PDAC Samples

**Figure 4:**
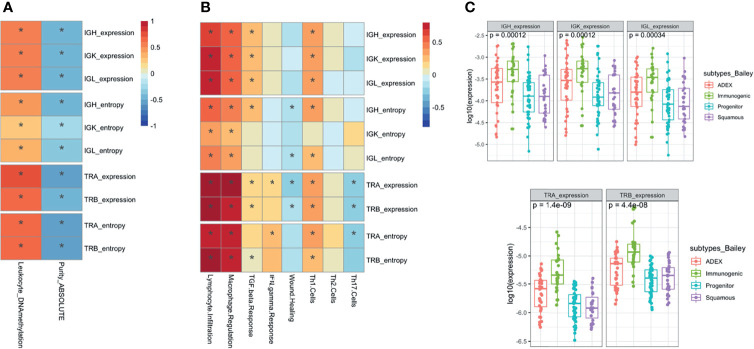
Association analysis with molecular and immune characteristics. Pearson correlation **(A)** with the association between the immune repertoire features and tumor purity and leukocyte DNA methylation measures. Pearson correlation **(B)** between the IG and TCR features with immune expression signatures and characteristics. The * shows if the correlation is significant (FDR < 0.05). Boxplots with the association between IG and TCR with subtypes of pancreatic cancer using a Wilcoxon test **(C)**.

We assessed the association between the IR features extracted from RNA with other characteristics extracted from other molecular data, such as somatic mutation and DNA methylation (23). We observed a significant inverse association between all the IG and TCR features (richness and diversity) with tumor purity measured using ABSOLUTE (7, 24), an approach that infers the purity based on DNA somatic mutations [correlation ranging from −0.3 (IGK entropy) to −0.6 (TRA expression) with very significant *p*-values from 0.001 to 3.3 × 10^−14^], and a positive association with leukocyte fraction based on DNA methylation as described by Carter et al. (24) [correlation ranging from 0.2 (IGK entropy) to 0.7 (TRA expression) with very significant *p*-values from 0.01 to 1.0 × 10^−23^] (**Figure 4A**). As expected, the tumors with higher IG or TCR infiltration were less pure and had more leukocytes based on DNA methylation data. We assessed IR features according to the PDAC subtype classification by Collison, Bailey, and Moffit and only observed differences in IR features between tumor subtypes based on Bailey’s classification: the immunogenic subtype showed the highest IG infiltration (*p* < 0.001 for IGs and *p* < 10^−8^ for TCRs) (**Figure 4C** and **Supplementary Figure S4**). Although significant, the lowest correlation with purity and DNA methylation was with the IGK entropy (**Figure 4A**), meaning that tumors that had lower diversity were purer and had less leukocytes based on DNA methylation.

Then, we characterized the IR by a distinct distribution of scores over five immune signatures and a set of compiled scores for Th1, Th2, and Th17 cells proposed by Thorsson et al. (10) (**Figure 4B**). As expected, we found a significant positive association between lymphocyte infiltration and levels of IG and TCR richness and diversity [correlation ranging from 0.4 (IGK entropy) to 0.9 (TRA expression) with highly significant *p*-values from 10^−6^ to 10^−45^ respectively]. Interestingly, we observed that macrophage regulation was also associated with higher levels of IG and TCR richness [correlation ranging from 0.3 (IGK entropy) to 0.8 (TRA expression) with highly significant *p*-values from 10^−4^ to 10^−29^ respectively]. More subtly, IG richness was associated with an increased level of TGF beta response [correlation 0.3 (*p* = 1.6 × 10^−3^), 0.2 (*p* = 1.1 × 10^−2^), and 0.2 (*p* = 8.4 × 10^−3^) for IGH, IGK, and IGL, respectively] while TCR diversity was associated with an increased level of IFN gamma response [correlation 0.2 (*p* < 10^−2^) TRA expression and entropy]. Moreover, TCR richness and diversity were associated with a decreased wound healing signature activity [correlation −0.3 (*p* = 2.5 × 10^−3^) TRA expression and 0.2 (*p* = 2.6 × 10^−2^) TRB expression], and the direction of the association between TCR and TGF beta response was negative.

In addition, IG and TCR richness were highly associated with increased levels of Th1 cells [correlation 0.3 (*p* < 10^−5^) IGH, IGK, and IGL expression 0.4 (*p* < 10^−5^) TRA and TRB expression and entropy], which are activators of both B and T cells in the immune system. Furthermore, a higher richness of TCR was associated with lower levels of Th17 [correlation −0.2 (*p* < 10^−2^) TRA and TRB expression].

### Immune Repertoire Features Were Associated With Clinical, Demographic, Tumoral Neoantigen Load and Mutation Rate, and Prognosis in PDAC Samples

**Figure 5:**
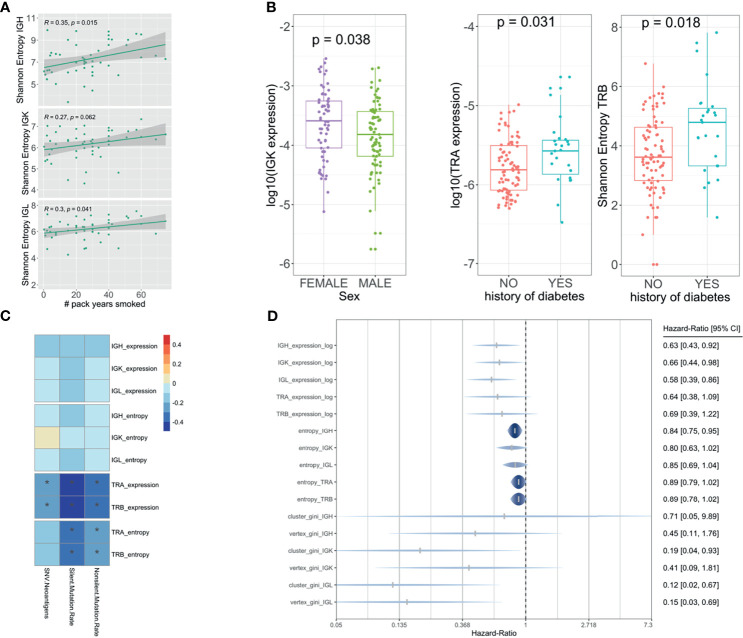
Association analysis with clinical, demographic, mutational rate, and prognosis. Linear regression model showing the association between the IG and smoking **(A)**. Boxplot showing the association between TCR and diabetes and the association between IG and sex using a Wilcoxon test **(B)**. Pearson correlation showing the association between the TCR and IG with neoantigen load and mutational rate **(C)**. Forest plot showing the hazard ratios and 95% confidence intervals for the Cox regression (adjusted by age, sex, and pathological stage) of IG/TCR measures with overall survival **(D)**. The * shows if the correlation is significant (FDR < 0.05).

We tested all the clinical and demographic variables available on the TCGA, and we found that IG richness and diversity were nominally significantly higher in females (*p* = 0.038 for IGK expression) and heavy smokers [*p* = 0.015, *p* = 0.062, and *p* = 0.041 (IGH, IGK, and IGL entropy) and *p* = 0.048, *p* = 0.031, and *p* = 0.031 (IGH, IGK, and IGL expression)] (**Figures 5A, B**) while TCR richness and diversity were associated with a history of diabetes [*p* = 0.031 and *p* = 0.0063 (TRA and TRB expression) and *p* = 0.026 and *p* = 0.018 (TRA and TRB entropy)] (**Figure 5B**). In addition, higher levels of TCR infiltration were associated (passed Bonferroni multiple testing correction) with a lower incidence of neoantigens [correlation −0.2 (*p* = 8.7 × 10^−3^) and −0.2 (*p* = 7.9 × 10^−4^), TRA and TRB expression] and silent [correlation −0.4 (*p* = 1.9 × 10^−7^), −0.3 (*p* = 6.6 × 10^−9^), −0.3 (*p* = 3.7 × 10^−5^), and −0.3 (*p* = 2.9 × 10^−5^), TRA and TRB entropy and expression] and non-silent mutational rate [correlation −0.3 (*p* = 3.1 × 10^−5^), −0.4 (*p* = 2.9 × 10^−6^), −0.3 (*p* = 8.3 × 10^−4^), and −0.3 (*p* = 5.3 × 10^−4^), TRA and TRB entropy and expression] (**Figure 5C**). We also performed a survival analysis using Cox regression analysis considering all IG and TCR measures adjusted for age, sex, and pathological stage. Higher richness, diversity, and clonal expansion for IGH (HR [95% CI] = 0.63 [0.43,0.92] and 0.84 [0.75,0.95], expression and entropy, respectively), IGK (HR [95% CI] = 0.66 [0.44,0.98] and 0.80 [0.63,1.02], expression and entropy, respectively), and IGL (HR [95% CI] = 0.58 [0.39,0.86] and 0.85 [0.69,1.04], expression and entropy, respectively) were associated with a better overall PDAC survival, while TCR was not (**Figure 5D**).

### IGK Clonotypes Distinguished PDAC Samples From Normal Pancreatic Tissue and Classified PDAC Patients With Better Prognosis Using Compositional Data Analysis

**Figure 6:**
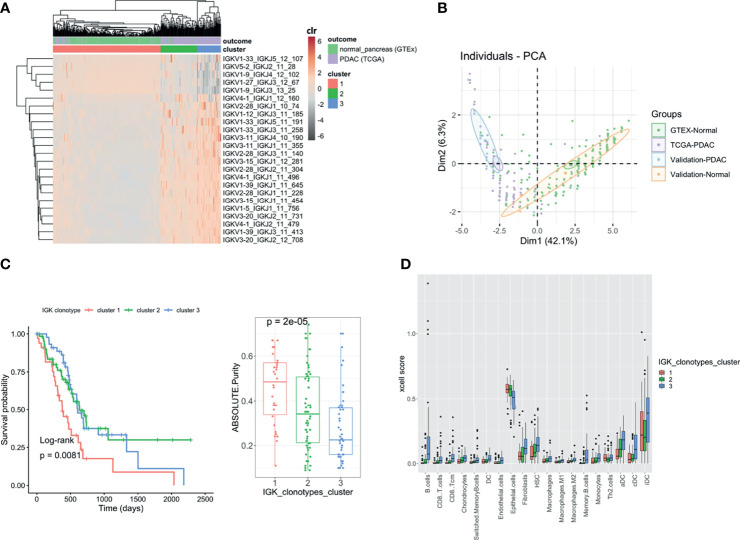
Clonotype analysis. Heatmap showing the IGK clonotypes selected by CLR-LASSO to discriminate PDAC (TCGA) *vs*. Normal Pancreas (GTEx). The three main clusters are obtained by unsupervised cluster analysis using hierarchical clustering **(A)**. Bi-plot showing the two first principal component analysis using the 24 clonotypes selected and applied to both discovery and validation datasets **(B)**. Kaplan–Meier curves for overall survival with the three clusters obtained and the boxplot with the tumor purity measured using ABSOLUTE^7,27^ that infers the purity based on DNA somatic mutations **(C)**. Comparison between the three clusters with the cell content deconvoluted using xCell. Those cells are the ones that show significant differences across the three clusters using Kruskal–Wallis test (FDR < 0.05) **(D)**.

We wanted to see whether some particular clonotypes (defined by the same V and J gene, same CDR3 length, and 90% nucleotide identity) were discriminating the PDAC samples from the normal pancreatic tissue. Clonotypes can be treated as compositional data (random vectors with strictly positive components whose sum is constant) since they are expressed as number of reads grouped by clones and are constrained by the sequencing depth. Therefore, to find whether particular clonotypes were associated with the outcome, we took advantage of the compositional data methodology that has been extensively used in microbiome studies. In this context, we applied the centered log ratio (CLR)-LASSO (25) to select the clonotypes associated with the outcome (normal *vs*. PDAC). We applied the CLR-LASSO to all IG chains, but we could not perform this analysis with TCR clonotypes due to the lack of overlapping clonotypes. We found 24 IGK clonotypes that discriminate PDAC from normal pancreas. Applying hierarchical clustering to those clonotypes, we found three main clusters that classified our PDAC samples (**Figure 6A**). Using principal component analysis (PCA), we validated the 24 clonotypes in the validation datasets as shown in **Figure 6B**, where both PDAC datasets were located together and both normal pancreas dataset clustered together away from the PDAC samples. Interestingly PDAC samples in cluster 1, which had lower infiltration and was more similar to the normal pancreas, were associated with higher purity and poor survival (**Figure 6C**). The clusters were not associated with sex, smoking, or history of diabetes. Finally, we performed a deconvolution analysis using xCell (26) and investigated whether there were differences in cell proportions among the three clusters (**Figure 6D**). We found that cluster 3 was the one with higher infiltration and better survival and had increased levels of DC, monocytes, all type of B cells, CD8 T cells, macrophages, HSC, and fibroblasts, as well as lower levels of epithelial cells.

### IGK Clonotypes Explained a Large Prognostic Phenotypic Variance

We finally obtained a prognostic score using all identified IGK clonotypes by applying a Bayesian Reproducing Kernel Hilbert Space regression (RKHS), where the kernel was linear (**Supplementary Figure S5**). We observed that the most similar samples based on their IGK profile were enriched with the ones previously clustered in cluster 3 using the 24 IGKs discriminating tumor and normal samples. On the contrary, samples with the lowest similarities based on their whole IGK profile were the ones belonging to previously defined cluster 1. After applying the RKHS adjusted by age, sex, and pathological stage, we estimated the variance explained by the IGK profiles, which explained 24% of the prognostic phenotypic variance.

## Discussion

We have conducted a systematic study to characterize the tumor-infiltrating B- and T-cell repertoire in PDAC and normal pancreatic samples. In normal tissues, the microenvironment represents an important barrier to avoid the development and spread of tumors. However, the pro-tumor immunity function in cancer cells would be altered by recruiting infiltrating immune cells transforming into tumor microenvironment benefitting tumor growth (32). It is the interaction and coevolution of cancer cells, as well as the microenvironment, that promotes the pancreatic tumor. In this analysis, we found that both richness and diversity of B- and T-cell IRs were higher in PDAC tumors compared to normal pancreatic tissue showing that TCR and IG may recognize a broad array of tumor-expressed and self-antigens.

We observed that all IGs and TCRs were positively correlated with lymphocyte infiltration and leukocyte DNA methylation as sources of proinflammatory mediators in the tumor microenvironment. They were also inversely correlated with tumor purity since tumor-infiltrating immune repertoire is associated with a low neoplastic cellularity. We also found that immunogenic and ADEX subtypes (8), previously associated with lower neoplastic cellularity (23), were associated with a higher tumor-infiltrating immune repertoire. These results were in line with previous work showing that the immunogenic subtype had a lower purity and higher leukocyte infiltration based on DNA methylation than the other subtypes (7) supporting the robustness of our IR data extracted from RNAseq. IG and TCR were positively associated with macrophage regulation pointing out to be regulators of tumor immunity (33). Nevertheless, TCR and IG seemed to have different patterns of activation and/or proliferation. TCRs were associated with increased levels of IFN-gamma response and lower levels of Th17 cells, in agreement with the fact that IFN-gamma is the main stimulator of Th1 differentiation and has been shown to inhibit Th17 differentiation. We also observed that tumor neoantigen load is inversely associated with lower TCR infiltration. In previous analysis of a different PDAC dataset, high mutational load has also been associated with fewer cytotoxic T cells (34). This inverse association, together with the fact that we found little evidence of expansion of the T-cell repertoires, suggests that infiltrating T cells are inactive/exhausted and they cannot generate an effective immune response. This could be due to an immune-suppressive tumor microenvironment and potential immune escape (35) decreasing the efficiency of neoantigen presentation in tumors where the T-cell infiltration is higher. Many human cancers express inflammatory molecules that lead to an intrinsic pro-oxidant environment in the cancer cells, as well as potentiating a microenvironment that drives immune escape and resistance to apoptosis.

We focused on the association between the IR and lifestyle risk factors and comorbidities. Indeed, we found that individuals with diabetes have higher TCR expression in their tumors. We could speculate whether the possible immune escape could be driven by some inflammatory pathways since diabetes is a pro-inflammatory PDAC risk factor (36). On the other hand, we observed that levels of IG were related with females and heavy smokers. Tobacco smoking might create a high frequency of somatic mutations, a huge burden of neoantigens, and an amplified immunogenicity. Indeed, smoking has been previously associated with higher infiltrated immune microenvironment in breast and lung cancer (37, 38). We observed borderline significance showing a higher neoantigens load among smokers in PDAC samples, but smoking has a lot of missing values and TCGA is not well designed for this purpose. Although the differences were nominally significant, we observed a higher amount of IGK expression in females. Similarly, a recent finding reported an increased expression of immune checkpoint genes, and those associated with B-cell recruitment and function, in high-grade bladder cancer tumors from females compared to those from males (39).

Interestingly, we observed approximately 40-fold differences between tumor and normal samples regarding the number of IG reads. The importance of B cells in the tumor microenvironment has been investigated and discussed recently, but it is still understudied in many tumors. Their role is diverse, being responsible of secretion of antibodies and cytokines, modulating T-cell and innate immune responses and recognizing antigens (40). B cells are described to affect pro- or anti-tumor response (18), and their role is still controversial, having been linked to both good and bad prognosis (41). In our analysis, we observed that IGs were clonally expanded in the tumor compared to normal pancreatic tissue and presumably active; thus, they could be playing an anti-tumor response since patients with more IG expression and diversity have a better prognosis, suggesting that an enhancement of these responses should be considered in the design of cancer immunotherapies.

CDR3 sequences derived from IGK are the most abundant across all tissues, accounting on average for 54% of the entire B-cell population (42). Interestingly, the IGK constant locus (IGKC) has been associated with an improved prognosis in colorectal cancer (43), non-small cell lung carcinoma (44), breast cancer (45), and ovarian cancer (46), suggesting the important role of the humoral immune system, especially the IGKC in cancer development. In this study, we observed a large presence of the IGK clonotypes to the PDAC development. First of all, IGK IRs showed larger differences in terms of clonal expansion compared to normal pancreatic tissue, and we were also able to classify the PDAC tumors in three main clusters based on a selection of 24 IGK clonotypes. These clusters displayed different prognosis outcomes. In addition, the cluster with the better prognosis was enriched in DCs, macrophages, and B and T cells, and had a lower tumor purity showing an active immune response, while the one with worse prognosis was enriched in epithelial cells and had higher purity representing the classical “tumor cell” subtype. Moreover, with a well-designed prognostic score using RKHS, we estimated that 24% of the prognostic phenotypic variance was explained by considering all IGK clonotypes showing a large association with this cluster system based on the similarity matrix obtained by linear kernel. In further studies, it would be interesting to examine whether the IGK clonotypes that infiltrate the PDAC tumor microenvironment could be found in blood or plasma and, consequently, they could be proposed as “non-invasive” prognosis biomarkers.

Several limitations of this study should be recognized. First, we extracted the IG and TCR from RNA-seq data. There are several tools, including MiXCR, that have been designed for this purpose and they provide accurate annotations, but future analysis with targeted sequencing and functional studies will be necessary to validate the extracted features and associations. Second, RNAseq data of pancreatic normal tissue and tumors were obtained separately from different studies, which could affect our results. However, our findings were also validated in completely independent datasets and demonstrate that the findings reported in this analysis are robust. We are aware that the validation set of normal pancreatic tissue is very small, but we are confident in the validation results since the two PDAC datasets behaved similarly in all the analysis performed. Nevertheless, these findings will need to be further validated in a larger sample size study and leveraging other complimentary approaches such as immunohistochemistry and/or targeted IR sequencing. In addition, another potential follow-up would be the comparison with adjacent normal matched samples, which would greatly enhance the biological and potential significance of the findings. Third, the sequencing depth was different within and across datasets. To address this issue, we used several strategies. We obtained the expression of IG and TCR by dividing the reads by their corresponding total sequencing depth for each sample. For the Shannon entropy and Gini index, we performed a subsampling sensitivity analysis to make sure that the results were accurate across all subsamples, and for the clonotype analysis, we used compositional data analysis using the CLR transformation. Finally, the clinical data provided by TCGA is limited since the purpose of the program initially was to characterize tumors at the molecular level; therefore, future analysis with more detailed clinical immune-related data, including biomarkers such as TLS, would be informative.

Despite these limitations, our results are sound, revealing that the tumor-infiltrating IR found in our study provide further insights necessary to understand the immunogenicity of PDAC. Being able to determine which PDAC cases have more infiltration of TCR and IG and which are the molecular and clinical factors associated with them will facilitate prevention of the disease and the development of potential therapeutical strategies and could be used to improve better patient stratification for clinical trials.

## Data Availability Statement

The raw data can be found at the legacy archive of the GDC (https://portal.gdc.cancer.gov/legacy-archive/search/f) and NCBI dbGaP (https://dbgap.ncbi.nlm.nih.gov/aa/wga.cgi?page=login). The processed data from MiXCR can be found in **Supplementary Data**.

## Ethics Statement

The studies involving human participants were reviewed and approved by TCGA data. The patients/participants provided their written informed consent to participate in this study.

## Author Contributions

NM, MS, and SP conceived the study design and analysis plan. SP and EL performed the data analysis. KY, IW, and AR extracted the data with MiXCR and helped in the data analysis. SP, NM, and MS supervised the work. All authors contributed to the article and approved the submitted version.

## Funding

The authors declare that this study received funding from AACR-AstraZeneca Immuno-oncology research fellowship. The funder was not involved in the study design, collection, analysis, interpretation of data, the written of this article or the decision to submit it for publication. This work was also partially supported by Fondo de InvestigacionesSanitarias (FIS), Instituto de Salud Carlos III, Spain (#PI18/01347), and Pancreatic Cancer Collective (PCC): Lustgarten Foundation & Stand-Up to Cancer (SU2C #6179).

## Conflict of Interest

The authors declare that the research was conducted in the absence of any commercial or financial relationships that could be construed as a potential conflict of interest.

## Publisher’s Note

All claims expressed in this article are solely those of the authors and do not necessarily represent those of their affiliated organizations, or those of the publisher, the editors and the reviewers. Any product that may be evaluated in this article, or claim that may be made by its manufacturer, is not guaranteed or endorsed by the publisher.

## Supplementary Material

Supplementary Figure 1 Diversity analysis in the subset of non-diabetic patients. Boxplots showing IGH, IGK, IGL, TRA and TRB expression **(A)** and Shannon entropy **(B)**. The p-values correspond to the statistical differences by applying a Wilcoxon-test. Correlation plot for the GTEX-normal dataset **(C)** and TCGA-PDAC dataset **(D)**. The numbers correspond to the Pearson coefficient and the colored ones are statistically significant (FDR < 0.05).

Supplementary Figure 2 Entropy estimation after applying a downsampling strategy for IG entropy **(A)** and TCR **(B)**. The estimates were calculated randomly sampled different proportions (20, 40, 60, and 80) of the sequence data for each sample, and them the entropy was calculated in its corresponding sampled dataset. The same processed was repeated 10 times in each proportion to avoid possible stochastic effects and calculated the mean value as the final estimate.

Supplementary Figure 3 Gini(V) and Gini(C) after applying a downsampling strategy. The estimates were calculated randomly sampled different proportions (20, 40, 60, and 80) of the sequence data for each sample and the entropy was calculated in its corresponding sampled dataset. The same processed was repeated 10 times in each proportion to avoid possible stochastic effects and calculated the mean value as the final estimate.

Supplementary Figure 4 Boxplots with the association between IG and TCR with subtypes of pancreatic cancer defined by Bailey, Collison, and Moffit.

Supplementary Figure 5 Similarity matrix between samples based on the IGK clonotypes calculated by linear kernel. IGK clonotypes based variance explained 24% of the prognostic phenotypic variance calculated using RKHS.
