# Supplementary figures and images for: Tumor-Infiltrating B- and T-Cell Repertoire in Pancreatic Cancer Associated With Host and Tumor Features
Source: Front Immunol. 2021 Sep 23;12:730746. doi: 10.3389/fimmu.2021.730746 (PMC8495220; doi:10.3389/fimmu.2021.730746)

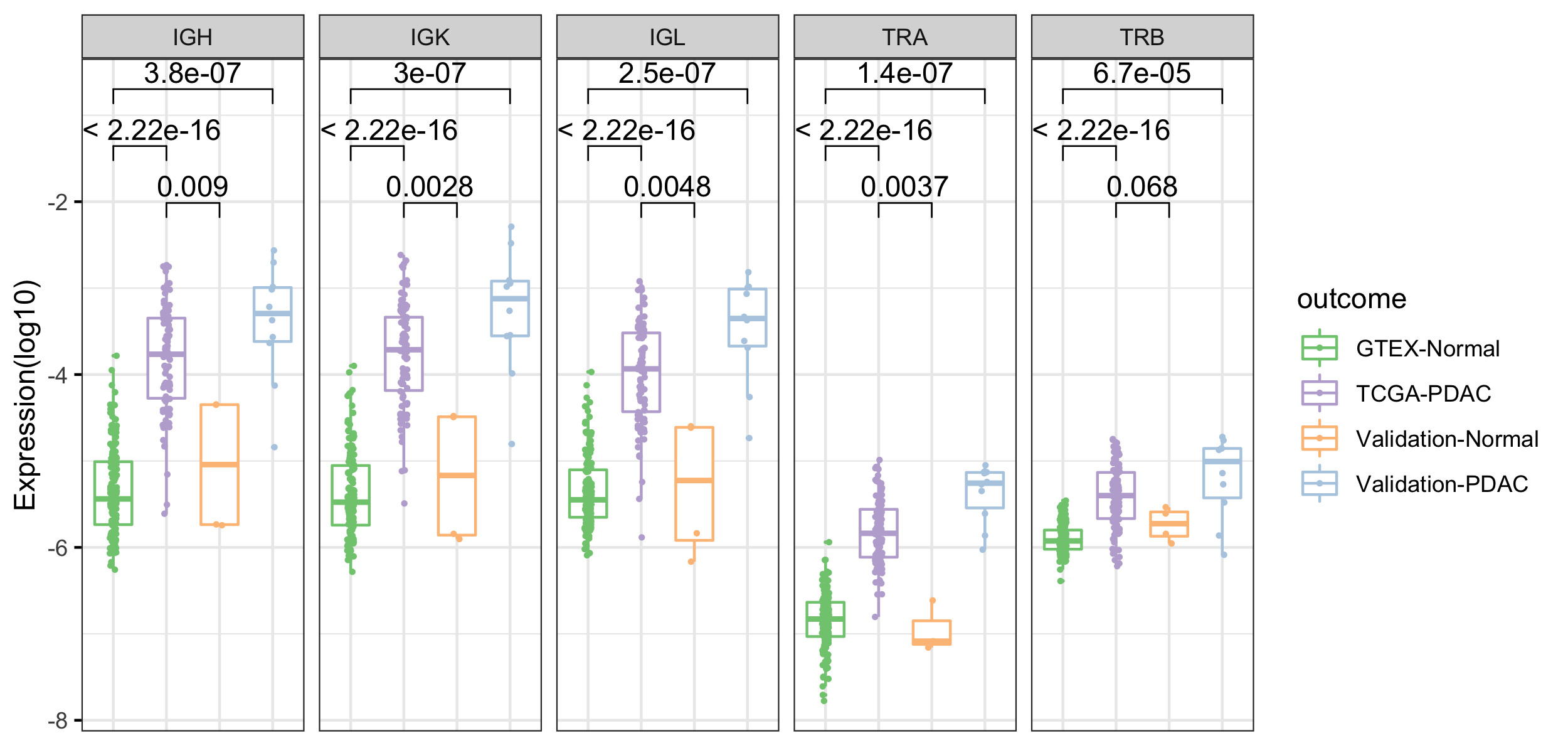

Supplement: Supplementary Figure 1 — Diversity analysis in the subset of non-diabetic patients. Boxplots showing IGH, IGK, IGL, TRA and TRB expression (A) and Shannon entropy (B). The p-values correspond to the statistical differences by applying a Wilcoxon-test. Correlation plot for the GTEX-normal dataset (C) and TCGA-PDAC dataset (D). The numbers correspond to the Pearson coefficient and the colored ones are statistically significant (FDR < 0.05). [file Image_1.tiff]

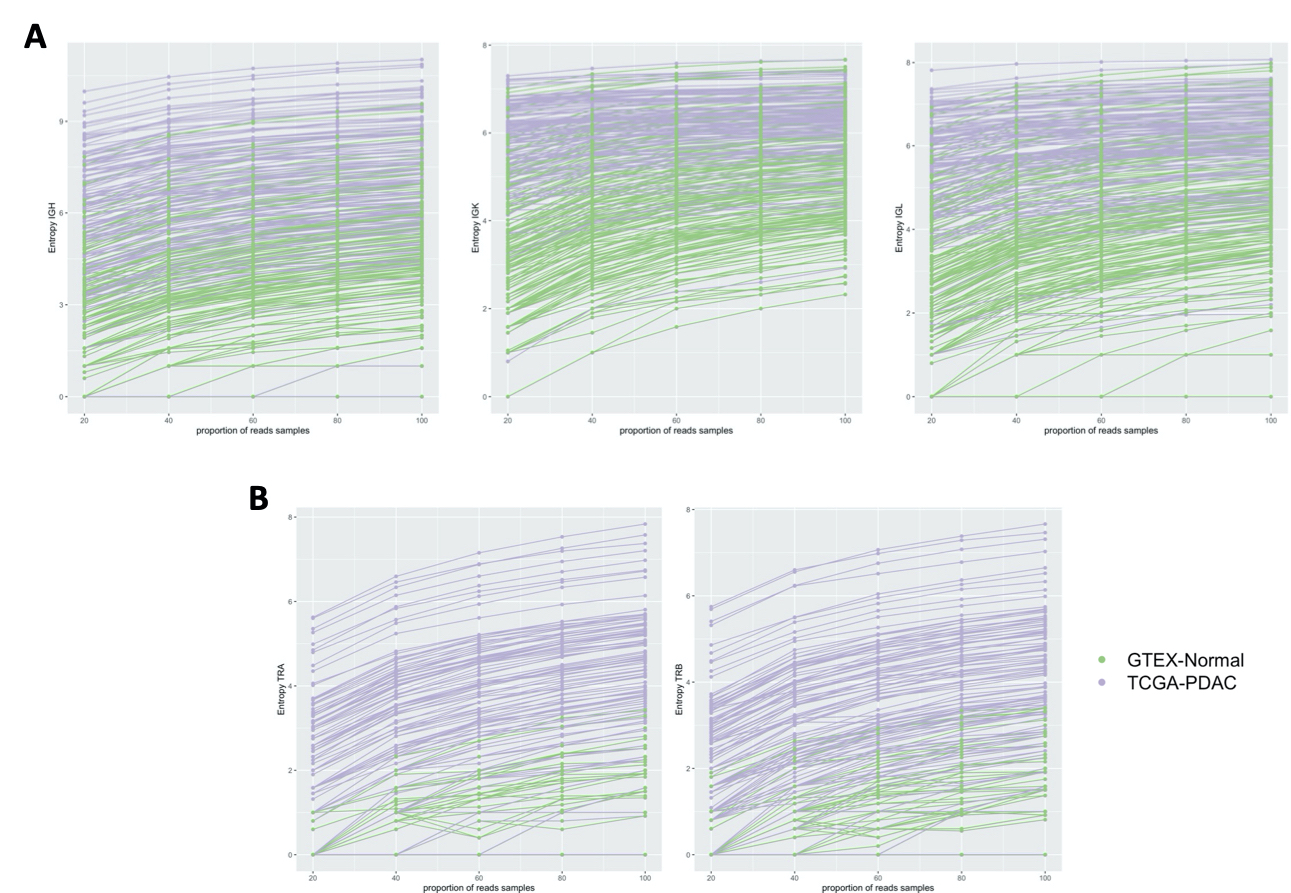

Supplement: Supplementary Figure 2 — Entropy estimation after applying a downsampling strategy for IG entropy (A) and TCR (B). The estimates were calculated randomly sampled different proportions (20, 40, 60, and 80) of the sequence data for each sample, and them the entropy was calculated in its corresponding sampled dataset. The same processed was repeated 10 times in each proportion to avoid possible stochastic effects and calculated the mean value as the final estimate. [file Image_2.jpeg]

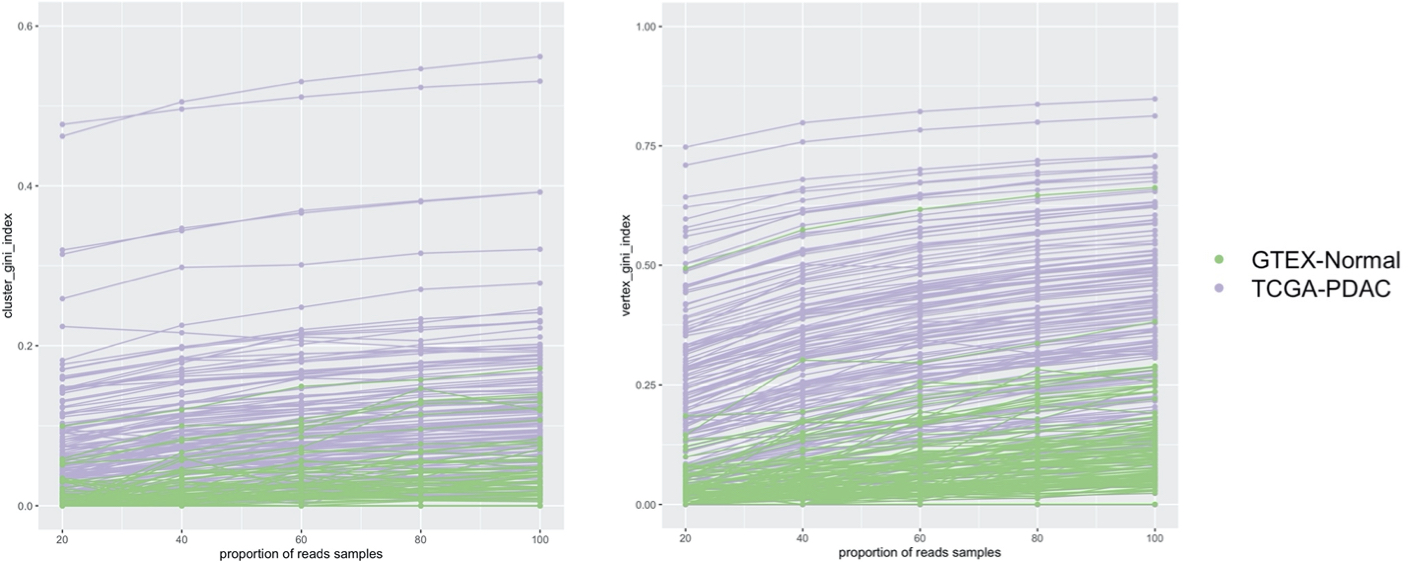

Supplement: Supplementary Figure 3 — Gini(V) and Gini(C) after applying a downsampling strategy. The estimates were calculated randomly sampled different proportions (20, 40, 60, and 80) of the sequence data for each sample and the entropy was calculated in its corresponding sampled dataset. The same processed was repeated 10 times in each proportion to avoid possible stochastic effects and calculated the mean value as the final estimate. [file Image_3.jpeg]

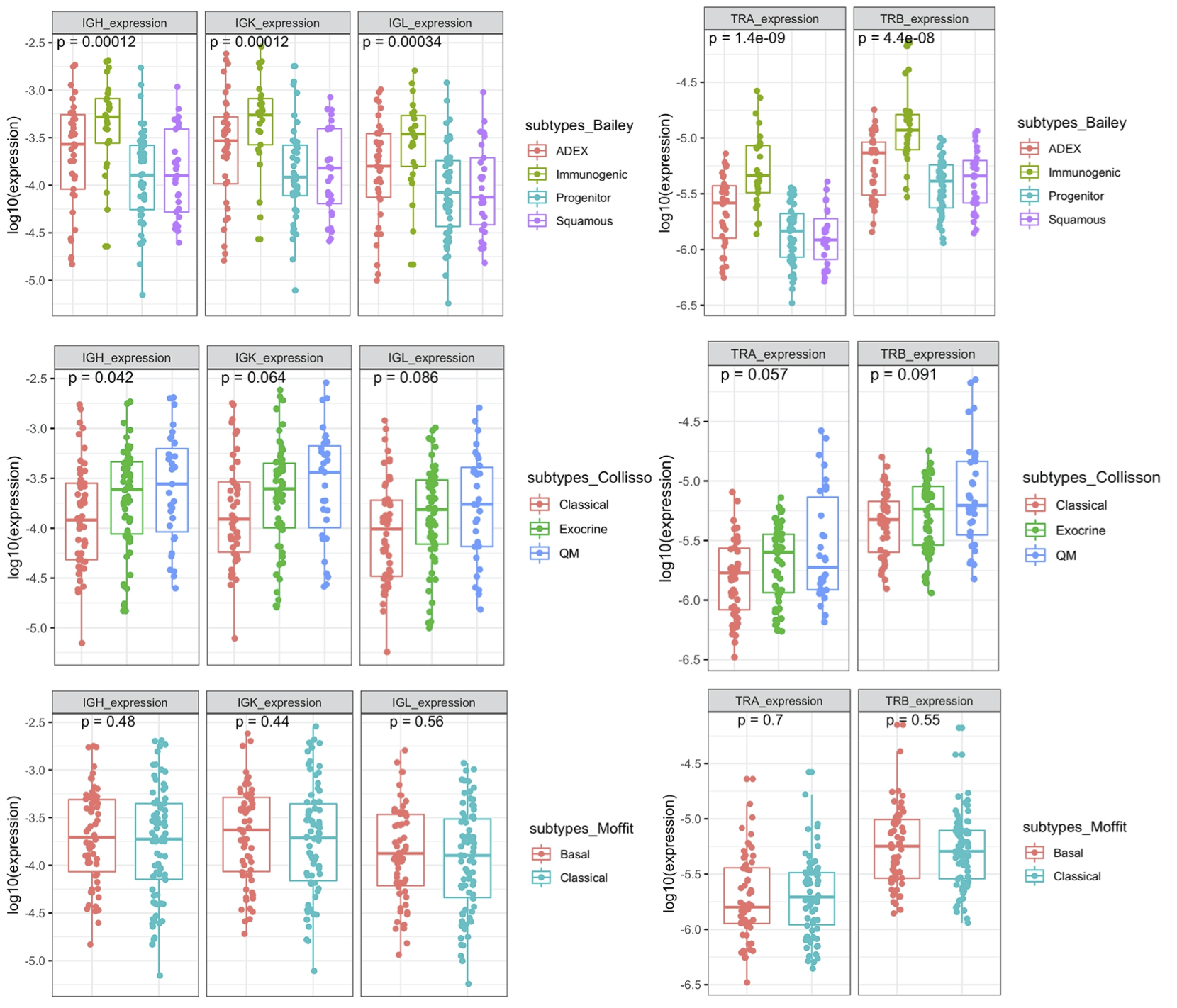

Supplement: Supplementary Figure 4 — Boxplots with the association between IG and TCR with subtypes of pancreatic cancer defined by Bailey, Collison, and Moffit. [file Image_4.jpeg]

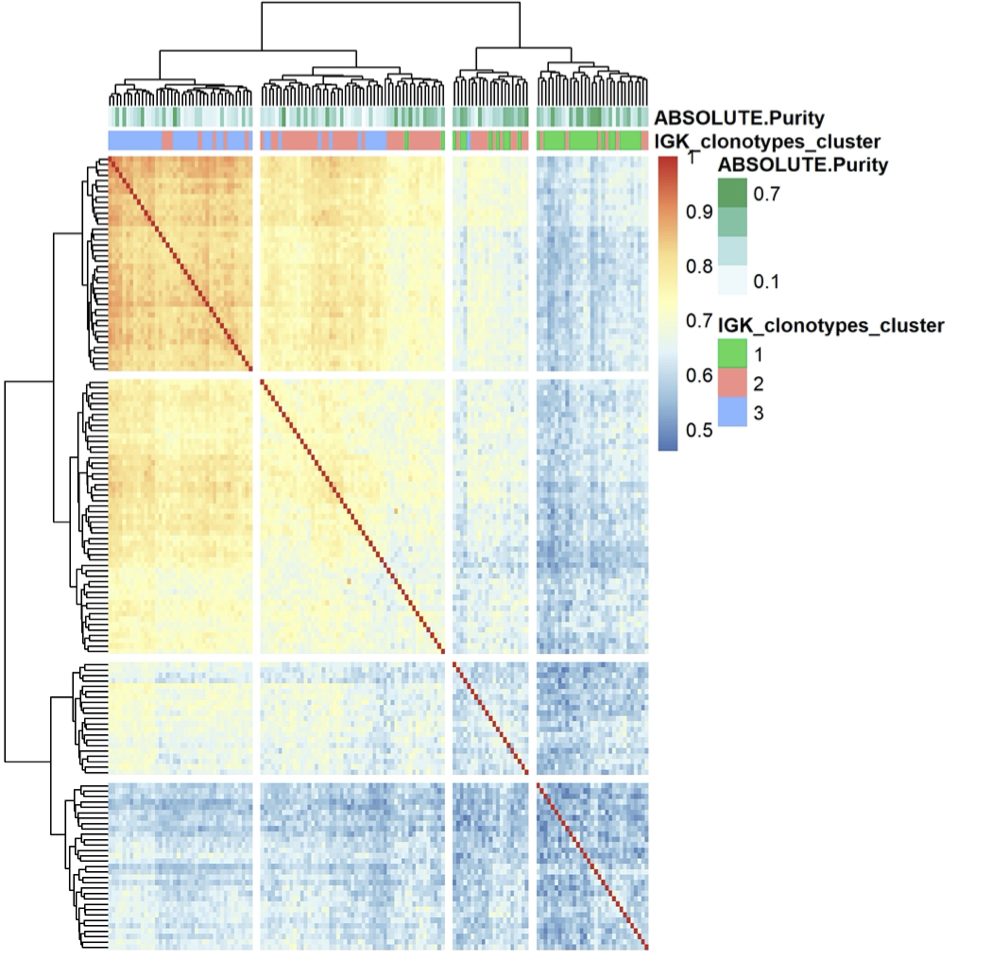

Supplement: Supplementary Figure 5 — Similarity matrix between samples based on the IGK clonotypes calculated by linear kernel. IGK clonotypes based variance explained 24% of the prognostic phenotypic variance calculated using RKHS. [file Image_5.jpeg]
