# Supplementary material for: Tumor-Infiltrating B- and T-Cell Repertoire in Pancreatic Cancer Associated With Host and Tumor Features
Source: Front Immunol. 2021 Sep 23;12:730746. doi: 10.3389/fimmu.2021.730746 (PMC8495220; doi:10.3389/fimmu.2021.730746)
Supplement: Supplementary file 7 [file Table_1.docx]

**Table S1. Sequencing Summary**

|  | **TCGA (PDAC)** | **Jie Lin et al. (PDAC)** | **GTEx (Pancreas)** | **Fagerberg Lin et al. (Pancreas)** |
| --- | --- | --- | --- | --- |
| **N** | **144** | **10** | **180** | **4** |
|  | **median (min-max)** |  |  |  |
| **IG reads**  **IGH**  **IGK**  **IGL** | 10,818 (54 – 341,320)  11,198 (88 – 283,164)  7,215 (56 – 171,217) | 20,121 (775 – 139,370)  25,862 (843 – 262,288)  15,985 (796 – 71,468) | 173 (18 – 6,230)  164 (17 – 5,870)  156 (28 – 4,168) | 285 (26 – 556)  206 (18 – 408)  160 (10 – 307) |
| **IG clones**  **IGH**  **IGK**  **IGL** | 320 (0 – 4,484)  271 (8 – 1,439)  240 (5 – 1,894) | 3,008 (145 – 10,933)  827 (130 – 3,433)  875 (109 – 3,377) | 22 (0 – 901)  38 (5 – 510)  28 (1 – 504) | 79 (8 – 154)  90 (10 – 179)  50 (3 – 104) |
| **TCR reads**  **TRA**  **TRB**  **TRD**  **TRG** | 105 (9 – 1,385)  295 (23 – 4,292)  4 (0 – 70)  10 (0 – 84) | 270 (17 – 505)  570 (44 – 993)  16 (1 – 67)  51 (2 – 117) | 7 (0 – 42)  52(14 – 159)  0 (0 – 5)  4 (0 – 21) | 1 (0 – 3)  25 (16 – 36)  0 (0 – 0)  0 (0 – 2) |
| **TCR clones**  **TRA**  **TRB** | 16 (0 – 243)  17 (0 – 257) | 108 (5 – 175)  135 (12 – 329) | 1 (0 – 11)  2 (0 – 15) | 0.5 (0 – 2)  2.5 (2 – 4) |
| **Total sequencing reads** | **122,076,385** (47,972,264 – 217,964,432) | **94,434,244**  (86,009,812 – 107,259,082) | **84,508,260**  (52,588,376 – 181,503,692) | **26,723,548**  (24,322,052 – 29,260,760) |
